# Gene co-expression analysis for functional classification and gene–disease predictions

**DOI:** 10.1093/bib/bbw139

**Published:** 2017-01-10

**Authors:** Sipko van Dam, Urmo Võsa, Adriaan van der Graaf, Lude Franke, João Pedro de Magalhães

**Affiliations:** 1 Department of Genetics, UMCG HPC CB50, RB Groningen, Netherlands; 2 Institute of Ageing and Chronic Disease, University of Liverpool, Liverpool, UK

**Keywords:** transcriptomics, functional genomics, disease gene prediction, next-generation sequencing, network analysis

## Abstract

Gene co-expression networks can be used to associate genes of unknown function with biological processes, to prioritize candidate disease genes or to discern transcriptional regulatory programmes. With recent advances in transcriptomics and next-generation sequencing, co-expression networks constructed from RNA sequencing data also enable the inference of functions and disease associations for non-coding genes and splice variants. Although gene co-expression networks typically do not provide information about causality, emerging methods for differential co-expression analysis are enabling the identification of regulatory genes underlying various phenotypes. Here, we introduce and guide researchers through a (differential) co-expression analysis. We provide an overview of methods and tools used to create and analyse co-expression networks constructed from gene expression data, and we explain how these can be used to identify genes with a regulatory role in disease. Furthermore, we discuss the integration of other data types with co-expression networks and offer future perspectives of co-expression analysis.

## Introduction

**Figure 1:**
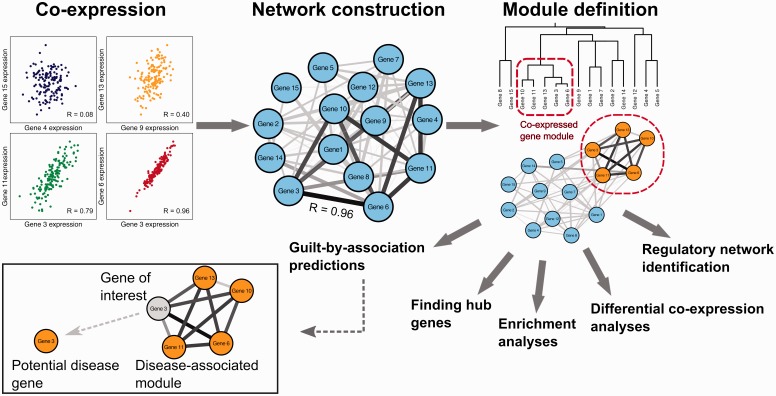
Example of a co-expression network analysis. First, pairwise correlation is determined for each possible gene pair in the expression data. These pairwise correlations can then be represented as a network. Modules within these networks are defined using clustering analysis. The network and modules can be interrogated to identify regulators, functional enrichment and hub genes. Differential co-expression analysis can be used to identify modules that behave differently under different conditions. Potential disease genes can be identified using a guilt-by-association (GBA) approach that highlights genes that are co-expressed with multiple disease genes.

A key objective in biological research is to systematically identify all molecules within a living cell and how they interact. However, the functions of many genes are still not understood, a situation that has only become more complex with the recent identification of many novel non-coding genes [1]. With the development of high-throughput technologies including microarrays and RNA sequencing (RNA-seq), and their respective data-analysis methods, the functional status of a gene can now be identified from a systematic perspective [2, 3]. One method to infer gene function and gene–disease associations from genome-wide gene expression is co-expression network analysis (Figure 1), an approach that constructs networks of genes with a tendency to co-activate across a group of samples and subsequently interrogates and analyses this network.

Gene co-expression networks can be used for various purposes, including candidate disease gene prioritization, functional gene annotation (Figure 1) and the identification of regulatory genes. However, co-expression networks are effectively only able to identify correlations; they indicate which genes are active simultaneously, which often indicates they are active in the same biological processes, but do not normally confer information about causality or distinguish between regulatory and regulated genes. An increasingly used method that goes beyond traditional co-expression networks is differential co-expression analysis [4–7]. This approach identifies genes with varying co-expression partners under different conditions, such as disease states [4, 8–10], tissue types [11] and developmental stages [12], because these genes are more likely to be regulators that underlie phenotypic differences. The regulatory roles of such genes can be further investigated by integrating data types such as protein–protein interactions, methylome data, interactions between transcription factors (TFs) and their targets, and with sequence motif analysis of co-expressed genes [13–15]. This aids in the identification of regulatory elements such as TFs, expression quantitative trait loci (eQTLs) and methylation patterns that affect the expression and composition of co-expression modules.

Gene expression and regulation can be highly tissue-specific, and most disease-related genes have tissue-specific expression abnormalities [16, 17]. The increased availability of expression data for multiple tissues has allowed for differential co-expression analysis, which can identify both tissue-specific signatures and shared co-expression signatures [11]. These tissue-specific signatures can be disrupted in tissue-specific diseases and would not be detected in analyses aggregating multiple tissues. Even when no sample classification is available, subpopulation-specific modules can be resolved, an approach that has been particularly successful in classifying different cancer subtypes to provide prognostic markers [18–20]. Differential co-expression analysis is also useful for analysing data sets in which the subpopulations are unknown, e.g. large-scale single-cell RNA-seq data [5, 12]. While differential co-expression methods are sensitive to noise [21], they are becoming more effective with the increase in RNA-seq data quantity and quality. RNA-seq further permits co-expression analysis to focus on splice variants and non-coding RNAs.

In this review, we provide an introduction and overview of what constitutes a co-expression network, followed by a guide of the different steps in co-expression analysis using RNA-seq data. We then describe commonly used and newly emerging methods and tools for co-expression analysis, with a focus on differential co-expression analysis to identify regulatory genes that underlie disease. We conclude with a discussion of the integration of co-expression networks with other types of data, to e.g. infer regulatory processes, and with future prospects and remaining challenges in the field.

## Co-expression networks

A co-expression network identifies which genes have a tendency to show a coordinated expression pattern across a group of samples. This co-expression network can be represented as a gene–gene similarity matrix, which can be used in downstream analyses (Figure 1). Canonical co-expression network construction and analyses can be described with the following three steps.

In the first step, individual relationships between genes are defined based on correlation measures or mutual information [22–24] between each pair of genes. These relationships describe the similarity between expression patterns of the gene pair across all the samples. Different measures of correlation have been used to construct networks, including Pearson’s or Spearman’s correlations [25, 26]. Alternatively, least absolute error regression [27] or a Bayesian approach [28] can be used to construct a co-expression network. The latter two have the added benefit that they can be used to identify causal links and have been explained elsewhere [29]. For a discussion of other types of similarity measures, we refer to [30]. Many of these similarity metrics can also be used to construct protein–protein interaction networks, which were compared using cancer data in [31].

In the second step, co-expression associations are used to construct a network where each node represents a gene and each edge represents the presence and the strength of the co-expression relationship (Figure 1) [32].

In the third step, modules (groups of co-expressed genes) are identified using one of several available clustering techniques. Clustering in co-expression analyses is used to group genes with similar expression patterns across multiple samples to produce groups of co-expressed genes rather than only pairs. The clustering method needs to be chosen with consideration because it can greatly influence the outcome and meaning of the analysis. Many clustering methods are available, including k-means clustering and hierarchal clustering, and are discussed in detail in [33]. Modules can subsequently be interpreted by functional enrichment analysis, a method to identify and rank overrepresented functional categories in a list of genes [34–36].

In co-expression analysis, it is important to consider the heterogeneity of the samples. Tissue-specific or condition-specific co-expression modules may not be detectable in a co-expression network constructed from multiple tissues or conditions because the correlation signal of the tissue/condition-specific modules is diluted by a lack of correlation in other tissues/conditions. However, limiting co-expression analysis to a specific tissue or condition also reduces sample size, thereby also decreasing the statistical power to detect shared co-expression modules. Therefore, methods that do not distinguish between tissues or conditions should be used for identification of common co-expression modules, while differential co-expression comparing different conditions or tissues will be better for identifying modules unique to a specific condition or tissue.

### Types of co-expression networks

#### Signed and unsigned co-expression networks

In a correlation-based co-expression network, correlation measures have values between −1 (perfect negative correlation) and 1 (perfect positive correlation). In an unsigned network, the absolute correlation values are used, which means that two negatively correlated genes will be considered as co-expressed. This causes negatively correlated genes to group together. Because those genes are likely to be also positively co-expressed with a completely different set of genes, these genes also group into the same module and disrupt the structure of the network. A signed network solves this problem by scaling the correlation values between 0 and 1 so that values <0.5 indicate negative correlation and values >0.5 indicate positive correlation. A signed method creates networks where biologically meaningful modules (such as those representing a specific biological process) are better separated [37]. Thus, a scaled value close to 0 indicates negative correlation, a feature which may be particularly interesting when microRNAs (miRNAs) are incorporated into the network, as these are known to exert their function mainly through down-regulation of other genes [38]. This also holds true for some long intergenic non-coding RNAs (lincRNAs) [39].

#### Weighted and un-weighted co-expression networks

In a weighted network, all genes are connected to each other, and these connections have continuous weight values between 0 and 1 that indicate the strength of co-regulation between the genes. In an un-weighted network, the interaction between gene pairs is binary, i.e. either 0 or 1, and genes are either connected or unconnected. An un-weighted network can be created from a weighted network by, for example, considering all genes with a correlation above a certain threshold to be connected and all others unconnected. We focus on weighted networks in this review because (to date) they have produced more robust results than un-weighted networks [40].

### Microarrays versus RNA-seq data

Co-expression networks can be constructed from gene expression data obtained from microarray or RNA-seq technology. One of the major benefits of RNA-seq is that it quantifies the expression of the over 70 000 non-coding RNAs not usually measured with microarrays [1], including recently annotated lincRNAs, many of which are thought to have regulatory roles [41] and to play a role in disease [42, 43]. Therefore, to gain a better understanding of the regulatory mechanisms driving biological processes, non-coding RNAs need to be considered in analyses.

RNA-seq also has other benefits [35]. It increases accuracy for low-abundance transcripts [44], has a higher resolution for identifying tissue-specific expression and distinguishes expression profiles of closely related paralogues better than microarray-derived profiles [45]. RNA-seq can also distinguish between the expression of different splice variants [46, 47], which can have distinct interaction partners [48] and biological functions [49]. Co-expression analysis on RNA-seq data can assign putative roles to these splice variants and lincRNAs [2], and identify diseases in which they might play a part [2]. A limitation of co-expression analysis on the splice variant level is the introduction of biases because it is difficult to determine which splice variant is expressed if multiple splice variants share the same expressed exon.

As an example of RNA-seq's utility with isoform- and exon-specific expression level measurements, exon-level expression was used to construct a co-splicing network [50, 51]. In a gene co-expression network, expression of different transcripts originating from the same gene is usually aggregated, which can lead to biased co-expression signals [50]. In a co-splicing network, this issue is resolved by considering the exon-expression-level distributions within a gene when calculating gene co-expression correlation. In biological terms this means that the expression of two genes is only considered to be correlated if their different splice variants show co-ordinated expression. If this is not the case, they are not considered to be co-expressed even if the overall expression levels of the genes are correlated. This approach has identified novel functional modules, which would not be detected using traditional co-expression networks [51]. Additionally, genes that contain multiple exons and transcripts acquired more relevant positions in the network using this method [50], a reassuring result given that splice variants can have different functions and are thus likely co-expressed with functionally distinct partners, which co-splicing networks account for.

A different approach is to determine the expression of different isoforms originating from the same gene based on the distributions of reads mapping to its various exons. This method is used by SpliceNet, which effectively divides the reads mapping to an exon shared with two isoforms proportionally to the total expression of each of the two whole isoforms [52]. This means that if two isoforms, isoform A and isoform B, share only one exon X (to which a number of reads map), but there are no reads mapping to the other exons of isoform A, whereas some reads map to the exons of isoform B, all reads mapping to exon X are then assigned to isoform B, resulting in isoform A being considered as not expressed at all. Although this elegant solution was validated using simulations, no experimental validation was conducted.

The most common way of constructing RNA-seq-based co-expression networks is to merge all overlapping gene isoforms in the RNA-seq data analysis and then construct the network at the gene level. This approach, however, loses information about different transcripts encoded by the same gene. Alternatively, transcript-based co-expression networks can be constructed. The drawback of these networks is their dramatic increase in size owing to the many gene isoforms and non-coding RNAs. As co-expression networks are square matrixes, the size of the network increases quadratically (n^2^) with the number of genes included. As there are ∼200 000 annotated transcripts in the human genome (according to Ensembl GRCh38.p5 (human) annotation [53]) and only ∼20 000 protein-coding genes, the resulting network increases 100-fold in size, greatly increasing the computational resources needed for the analysis. One solution to this problem is to build co-expression network blocks from subsets of the data and combine these blocks at a later point in the analysis [54]. We recommend users to be cautious with block-wise clustering, however, as it may influence the results of subsequent module detection analyses, and it is unclear how well these perform when large numbers of blocks are used.

### RNA-seq data for co-expression networks

RNA-seq analysis entails multiple steps that include obtaining expression estimates from the sequenced reads, data normalization and quality control. Different tools and methods to obtain reliable expression counts from RNA-seq data were recently reviewed in [55], and these will not be reviewed here.

In our experience, different normalization methods introduce different biases in co-expression analysis, usually towards positive correlation. New methods are continuously being created to tackle these normalization issues. The recently published method extracting patterns and identifying co-expressed genes (EPIG) from RNA-Seq data (EPIG-seq), for example, is designed to calculate gene correlation across RNA-seq samples, being unaffected by read-depth differences between samples and the large abundance of 0 values present in RNA-seq-derived expression matrices [56]. Biases originating from the large abundance of 0 values are even more pronounced in single-cell experiments because of low RNA quantities per cell. Specific tools have been created for analysis of single-cell RNA-seq data and are reviewed in [57]. Although some studies comparing different normalization methods for RNA-seq data are available [58], more comprehensive comparison studies incorporating newer methods are needed.

#### Minimum read depth and sample size required for co-expression analyses

To create co-expression networks from RNA-seq data, a 20-sample minimum has been suggested [21, 54], and increased sample sizes produce networks with a higher functional connectivity [21, 59]. Not surprisingly, higher quality data tend to result in more accurate co-expression networks [21, 59]. It is therefore essential to set cut-off thresholds for data quality control. A higher total read depth for RNA-seq samples increases the accuracy of the expression measurements, especially for genes with low expression [21, 59]. For RNA-seq data, sequencing depth cut-off thresholds are usually selected arbitrarily. Several co-expression studies have used a cut-off of 10 million reads per sample [2, 21, 60]. Co-expression networks constructed using this cut-off have been suggested to have a similar quality to microarray-based co-expression networks if constructed from the same number of samples [21], but decreasing in quality with fewer reads. The percentage of mapped reads is another frequently considered cut-off in which samples with <70% or 80% of the reads mapping to the genome are removed. Giorgi et al. demonstrated, using 65 *Arabidopsis thaliana* samples with 12 million reads but applying only a 30% mapping cut-off threshold, that the resulting RNA-seq-based co-expression network had a lower similarity to biological networks than microarray networks [61]. Cut-off thresholds may vary per species, based on, among other factors, the quality of the genome annotation. As more and higher quality data become available, higher cut-off thresholds may be preferable.

To ensure that a network is robust, bootstrapping can be used [62]. This is the repetitive construction of networks by using random sets of samples (one sample can be part of multiple subsets) from the data, which are subsequently used to assess the reproducibility of the network created from the entire data set. Randomizing the data set (e.g. by randomly reassigning expression values to their gene/transcript identifiers and reconstructing the network) can also help identify correlations that occur stochastically because of specific biases rather than as a result of biologically relevant interactions [2].

## Clustering and network analysis

### Identifying modules

Clustering is used to group genes that have a similar expression pattern in multiple samples. The resulting modules often represent biological processes [63, 64] and can be phenotype specific [65].

The most widely used clustering package for co-expression analysis is Weighted Gene Correlation Network Analysis (WGCNA) [40]. This easy-to-use tool constructs co-expression modules using hierarchical clustering on a correlation network created from expression data [54]. Hierarchical clustering iteratively divides each cluster into sub-clusters to create a tree with branches representing co-expression modules. Modules are then defined by cutting the branches at a certain height (Figure 1).

WGCNA was the first co-expression tool to be applied to RNA-seq data; it has effectively identified biologically relevant associations between phenotypes and modules [19, 66, 67], performing similarly to microarray-based analyses. An RNA-seq-based co-expression study on normal and failing murine hearts found that many lincRNAs are present in clusters correlating with the failing murine heart phenotype, suggesting a possible role of these non-coding RNAs in this disease [67]. Co-expression analysis of RNA-seq data of lean and obese porcines identified obesity-related modules [66], and a link was found between obesity, the immune system and bone remodelling, with the study identifying *CCR1*, *MSR1* and *SPI1* as possible regulators in these processes. WGCNA was also used to identify biologically relevant associations from single-cell RNA-seq data. Regulatory mechanisms and genes underlying pre-implantation processes conserved between humans and mice were identified by using preservation detection defined by WGCNA [12], a feature that was later added to this package [68]. Co-expression modules were identified for different developmental stages of human and mice separately. The modules identified for each stage were then compared between humans and mice to reveal a strong overlap between co-expression modules in oocyte formation in mice and oocyte and single-cell stage co-expression modules in humans. This suggests that humans and mice share core transcriptional programmes in early development, but diverge at a later stage [12].

### Identifying hub genes

**Figure 2:**
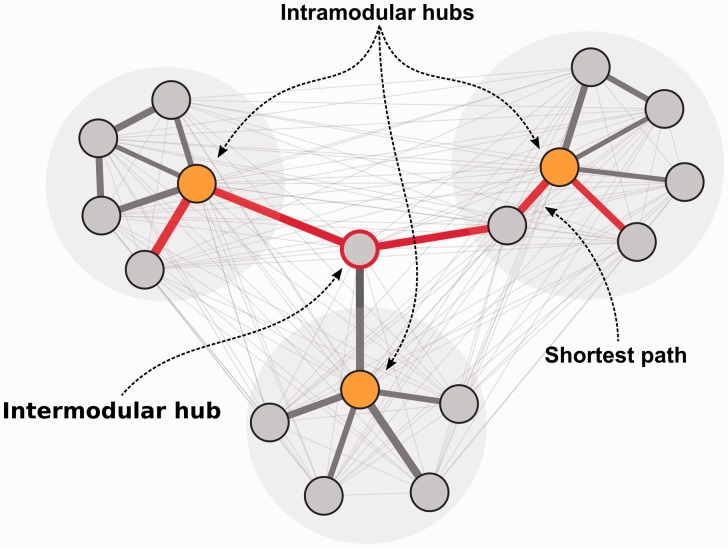
Hypothetical network explaining inter- and intra-modular hubs and network centrality. The inter-modular hub has a high network centrality, as it is required for the largest number of shortest paths between all possible node pairs. The red line indicates an example of a shortest path through the network between a pair of nodes. Intra-modular hubs (marked with orange) are central to individual modules and usually have high biological relevance.

Co-expression modules identified by clustering are often large, and so, it is important to identify which gene(s) in each module best explains its behaviour. A widely used approach is to identify highly connected genes in a co-expression network (hub genes). Hubs are frequently more relevant to the functionality of networks than other nodes [69]. This is also the case in biological networks [32], although mathematical derivations show that this is only the case for intra-modular hub genes (as opposed to inter-modular hub genes [64, 65]). Intra-modular hubs are central to specific modules in the network, while inter-modular hubs are central to the entire network (Figure 2). To identify hub genes, centrality measures, mainly ‘betweenness centrality’, are often used. Genes with high betweenness centrality are important as shortest-path connectors through a network [70]. Connectivity is often used to measure network robustness and indicates how many genes need to be removed from the network before the remaining genes are disconnected. Identifying hub genes in co-expression networks has led to the identification of several genes essential in cancer [71, 72], type 2 diabetes [73], chronic fatigue [74], other diseases [75, 76] and tissue regeneration [77].

As there are usually multiple hubs or differentially connected genes in a module, it is not always clear which is the most important gene underlying a phenotype. Nor is there a guarantee that any of the hub genes is causal to a phenotype [78]. One study using myocardial data from 1617 samples found that known foetal gene markers upregulated in gene networks common to developing and diseased myocardium were not hub genes [79]. Another co-expression study in *Salmonella* found that hub genes are dispensable for growth, stress adaptation and virulence, suggesting that hub genes are not necessarily essential [80].

### Guilt by association

A widely used approach to attach biological meaning to modules is to determine functional enrichment among the genes within a module using e.g. the tools described in Table 1. Assuming that co-expressed genes are functionally related, enriched functions can be assigned to poorly annotated genes within the same co-expression module, an approach commonly referred to as ‘guilt by association’ (GBA) [121]. GBA approaches are also widely used to identify new potential disease genes if a substantial proportion of the genes within a module are associated with a particular disease [26, 121–126] (Figure 1).

**Table 1:** Methods and tools for RNA-seq-based co-expression network analysis

Tool/method	Description, strengths (+) and limitations (−)
Quality control	
FastQC [81] http://www.bioinformatics.babraham.ac.uk/projects/fastqc/	• A tool that uses .fastq, .bam or .sam files to identify and highlight potential issues in the data, such as low base quality scores, low sequence quality and GC content biases.
	+ Can be used either with or without user interface.
	− Uses only the first 200 000 sequences in the file.
RSeQC [82] http://rseqc.sourceforge.net/	+ A tool with a wider range of quality control measures than FastQC.
	+ Can also be used on mapped data to obtain information on metrics such as the prevalence of splicing events.
QoRTs [83] http://hartleys.github.io/QoRTs/	+ This is a similar tool to RSeQC but incorporates more quality control metrics.
Read Mappers	
Bowtie/Tophat/Tophat2 [84] https://ccb.jhu.edu/software/tophat/index.shtml	• The first widely used mapping tool.
	+ Detects splice variants.
	− Currently much slower than most other mappers and requires a relatively large amount of memory.
STAR [85] https://code.google.com/p/rna-star/	• A widely used tool to align reads to a genome.
	+ Maps ∼50 times faster than Tophat and Tophat2.
	+ Commonly used tool to detect novel splice variants.
	− Uses a large amount of memory (>20 GB for mapping to the human genome).
HISAT [86] http://www.ccb.jhu.edu/software/hisat/index.shtml	• A widely used tool to align reads to a genome at a faster rate than STAR with comparable accuracy.
	+ HISAT2 is expected to be the core of the next version of Tophat (Tophat3).
	+ Detects novel splice variants.
	+ The newer HISAT2 version aligns to genotype variants, likely achieving higher accuracy.
	+ Uses less memory than STAR (<8 GB for mapping to the human genome using default settings).
BWA [87]	• A commonly used aligner for species in which splicing does not occur.
	− Does not detect splice variants.
Kallisto [88] https://pachterlab.github.io/kallisto/about.html	• A tool that uses a pseudoalignment strategy to assign expression values to transcripts/genes to achieve optimal speed.
	• Comparable accuracy to other tools using real alignment strategies.
	• Reports reads/expression per gene instead of read alignment coordinates (which are commonly used to acquire the expression per gene).
	+ Uses little memory and can be run on a regular desktop computer.
	− Does not identify novel splice variants
Salmon [89] http://combine-lab.github.io/salmon/	• Another pseudoalignment tool. Performance comparable with Kallisto.
	• Reports reads/expression per gene instead of read alignment coordinates (which are commonly used to acquire the expression per gene).
	− Does not identify novel splice variants.
Read counting tools	
HTseq [90] http://www-huber.embl.de/HTSeq/doc/overview.html	• A tool that assigns expression values to genes based on reads that have been aligned with, e.g. STAR or HISAT.
	+ Well documented and supported.
FeatureCounts [91] http://bioinf.wehi.edu.au/featureCounts/	+ A tool that is similar to HTseq but much faster. Results are slightly different owing to slightly different expression assignment strategies.
SpliceNet [52] http://jjwanglab.org/SpliceNet/	• A tool that divides the reads mapping to an exon shared with two isoforms proportionally to the total expression of each of the two whole isoforms.
	+ Estimates expression more accurately when multiple genes/transcripts partly share the same genome regions.
Normalization	
FPKM/RPKM [92]	• Widely used normalization methods that correct for the total number of reads in a sample while accounting for gene length.
	− TMM has been suggested as a better alternative [58].
TPM [93]	• A method similar to FPKM, but normalizes the total expression to 1 million, i.e. the summed expression of TPM-normalized samples is always 1 million.
TMM [94]	• Similar to FPKM/RPKM but puts expression measures on a common scale across different samples.
RAIDA [95]	• A method that uses ratios between counts of genes in each sample for normalizations.
	+ Avoids problems caused by differential transcript abundance between samples (resulting from differential expression of highly abundant gene transcripts).
DEseq2 [96]	• A normalization method that adjusts the expression values of each gene in a sample by a set factor. This factor is determined by taking the median gene expression in a sample after dividing the expression of each gene by the geometric mean of the given gene across all samples. This differs from the normalization implemented in the DEseq2 differential expression analysis.
	• Implemented into the DEseq2 R package.
Correction for batch effects	
Limma-removeBatchEffect [97]	• A method which uses linear models to correct for batch effects.
Svaseq [98] https://github.com/jtleek/svaseq	• This method estimates biases based on genes that have no phenotypic expression effects, which are then used for correction of the data.
	• Specifically designed for RNA-seq data.
Combat [99] http://www.bu.edu/jlab/wp-assets/ComBat/Abstract.html	• A method that is robust to outliers and also effective at batch effect correction in small sample sizes (<25).
Co-expression module detection	
WGCNA [54] https://labs.genetics.ucla.edu/horvath/CoexpressionNetwork/Rpackages/WGCNA/	• A tool that constructs a co-expression network using Pearson correlation (default) or a custom distance measure.
	• Uses hierarchical clustering and has various ‘tree cutting’ options to identify modules.
	+ Most widely used tool, well supported and documented.
DiffCoEx [100]	• A method that uses a similar approach to WGCNA to identify and group differentially co-expressed genes instead of identifying co-expressed modules.
	• Identifies modules of genes that have the same different partners between different samples.
DICER [4]	• A method that identifies modules that correlate differently between sample groups, e.g. modules that form one large interconnected module in one group compared with several smaller modules in another group.
CoXpress [101] http://coxpress.sourceforge.net/	• A tool that identifies co-expression modules in each sample group and tests whether the genes within these modules are also co-expressed in other groups.
DINGO [102]	• DINGO is a more recent tool that groups genes based on how differently they behave in a particular subset of samples (representing e.g. a particular condition) from the baseline co-expression determined from all samples
GSCNA [103]	• A tool that tests whether a predefined defined gene set is differentially expressed between two sample groups.
GSVD [104]	• A method that identifies ‘genelets’, which can be interpreted as modules representing partial co-expression signals from multiple genes. These signals are then compared between two groups to identify genelets unique to samples and genelets that are shared between the two groups.
HO-GSVD [105] https://github.com/aanchan/hogsvd-python/blob/master/README.md	• A tool similar to GSVD, but that can be used across multiple sample groups rather than only two.
Biclustering [106]	• A group of methods that identify modules that are unique to a subpopulation of samples without the need for prior grouping of samples.
Functional enrichment	
DAVID [107] https://david.ncifcrf.gov/	• A widely used tool with an online web interface. Users supply a list of genes and select the annotation categories from various sources to identify enrichment.
PANTHER [108] http://pantherdb.org/	• A tool that uses a comprehensive protein library combined with human curated pathways and evolutionary ontology.
	• If a gene is not in the library, it is classified based on its protein sequence conservation and by finding a related gene.
g:Profiler [109] http://biit.cs.ut.ee/gprofiler/	• A tool that performs enrichment analyses for gene ontologies, KEGG pathways, protein–protein interactions, TF and miRNA binding sites.
	+ Also available as an R package.
ClusterProfiler [110] https://github.com/GuangchuangYu/clusterProfiler/blob/master/vignettes/clusterProfiler.Rmd	• An R package for overrepresentation and gene set enrichment analyses for several curated gene sets.
	+ Allows users to compare the results of analyses performed on several gene sets.
Enrichr [111] http://amp.pharm.mssm.edu/Enrichr/	• An intuitive web tool for performing gene overrepresentation analyses using a comprehensive set of functional annotations.
ToppGene [36] https://toppgene.cchmc.org/	• An intuitive tool that determines enrichment of different categories such as GO terms, chromosomal locations and disease associations.
	+ Also has other functions, such as candidate gene prioritization, based on network structures.
Regulatory network inference	
ARACNE [112]	• A tool that removes indirect connections between genes (i.e. partners of a gene that have a stronger correlation with each other than with the gene itself), leaving only those connections that are expected to be regulatory.
	+ Creates directional networks.
Genie3 [113]	• A tool that incorporates TF information to construct a regulatory network by determining the TF expression pattern that best explains the expression of each of their target genes.
	+ Creates directional networks.
	− Requires TF information.
CoRegNet [114]	• A tool that identifies co-operative regulators of genes from different data types.
cMonkey [115]	• Calculates joint bicluster membership probability from different data types by identifying groups of genes that group together in multiple data types.
Visualization	
Cystoscape [116] http://www.cytoscape.org/	• A widely used tool for the visualization of networks.
	+ Has many plug-ins available for specific analyses.
BioLayout [117] http://www.biolayout.org/	• Similar to Cytoscape but less widely used.
	+ Can load and visualize much larger networks than Cytoscape.
Co-expression databases^a^	
COXPRESdb [60] http://coxpresdb.jp/	• A web resource incorporating 12 co-expression networks for different species created from ∼157 000 microarrays and 10 000 RNA-seq samples. Has a focus on protein-coding RNAs.
GeneFriends [2] http://www.genefriends.org/	• Human and mouse gene and transcript co-expression networks.
	• Networks constructed from ∼4000 RNA-seq samples each.
	+ Includes a number of non-coding RNAs (∼10 000 for mouse and ∼25 000 for human).
GeneMANIA [118] http://www.genemania.org/	• Also includes physical and genetic interaction, co-localization, pathway and shared protein domain information data sets.
	+ Networks for nine species.
GENEVESTIGATOR [119] https://genevestigator.com/gv/	• A database constructed using ∼145 000 samples.
	+ Curated database.
	+ Networks for 18 species.
	+ Multiple data types.
GIANT [120] http://giant.princeton.edu/	• Tissue-specific interaction network database.
	• Includes 987 Datasets encompassing 38 000 conditions describing 144 tissues types.
	+ Integrates physical interaction, co-expression, miRNA binding motif and TF binding site data.

This is a non-comprehensive list of available tools and methods.

^a^These databases can be queried for a gene or multiple genes of interest to identify commonly co-expressed genes across the samples the database was created from.

When using a GBA approach it is important to remember that not every gene in a module necessarily correlates with a function or disease association for which it is enriched. Because co-expression modules often consist of a large number of genes, any overrepresentation of a functional process or group of disease-associated genes quickly becomes statistically significant, as often indicated by deceivingly low *p*-values. Misinterpretation of these low *p*-values may lead to the incorrect conclusion that all genes in a module play an important part in a particular process or disease. In reality, the fraction of genes in a module that relate to its main biological function is often <20% [127], and module-trait correlations can be relatively low (correlation < 0.5) even when statistically significant [128].

#### Regulatory network construction

Although there is ample evidence that co-expression analysis can help identify genes that play an important role in disease and biological functions, it remains difficult to infer causality from co-expression networks. Tools such as ARACNE [23] and GENIE3 [113] attempt to construct regulatory networks from co-expression networks. ARACNE removes indirect connections between genes (i.e. partners of a gene that have a stronger correlation with each other than with the gene itself), leaving only those connections that are expected to be regulatory. GENIE3 incorporates TF information to construct a regulatory network by determining the TF expression pattern that best explains the expression of each of their target genes. A limitation of GENIE3 is that TF information is required for it to perform better than random chance [113]. The performance of these methods has been compared with gold standards defined by regulatory interactions experimentally validated in >150 studies. The comparison suggests that methods attempting to derive regulatory networks from co-expression networks alone can only reliably distinguish between true- and false-positive regulatory interactions if perturbation experiment data are used for network construction [129]. A comparison between these tools and others, including WGCNA, showed that WGCNA and ARACNE perform best at defining the network structure of *Escherichia coli* [130], for which a well-defined regulatory network was used as a gold standard [131].

## Differential co-expression analysis

Differential co-expression analysis can identify biologically important differential co-expression modules that would not be detected using regular co-expression or differential expression analyses. Genes that are differentially co-expressed between different sample groups are more likely to be regulators, and are therefore likely to explain differences between phenotypes [4, 8–10]. Differential co-expression analysis has been used to identify genes underlying differences between healthy and disease samples [4, 8–10] or between different tissues [11], cell types [5] or species [132, 133]. Below, we provide an overview of commonly used and newly emerging methods and tools, separated into two categories: (1) approaches that identify differential co-expression between predefined sample groups (such as conditions, time points or tissue types) and (2) approaches that do not require prior knowledge about sample groups and use an algorithm that identifies co-expression clusters in a priori unknown subpopulations of the samples.

### Differential co-expression analysis between sample groups

Most differential co-expression analyses rely on differential clustering; they identify clusters that contain different genes or behave differently under changing conditions or phenotypes. The most frequently used programs for differential clustering analysis, which have also been compared with others programs, are WGCNA [54], DICER [4] and DiffCoEx [100], all of which first identify modules co-expressed across the full set of study samples. These co-expressed modules can then be correlated to predefined sample subpopulations representing, for example, disease status or tissue type.

**Figure 3:**
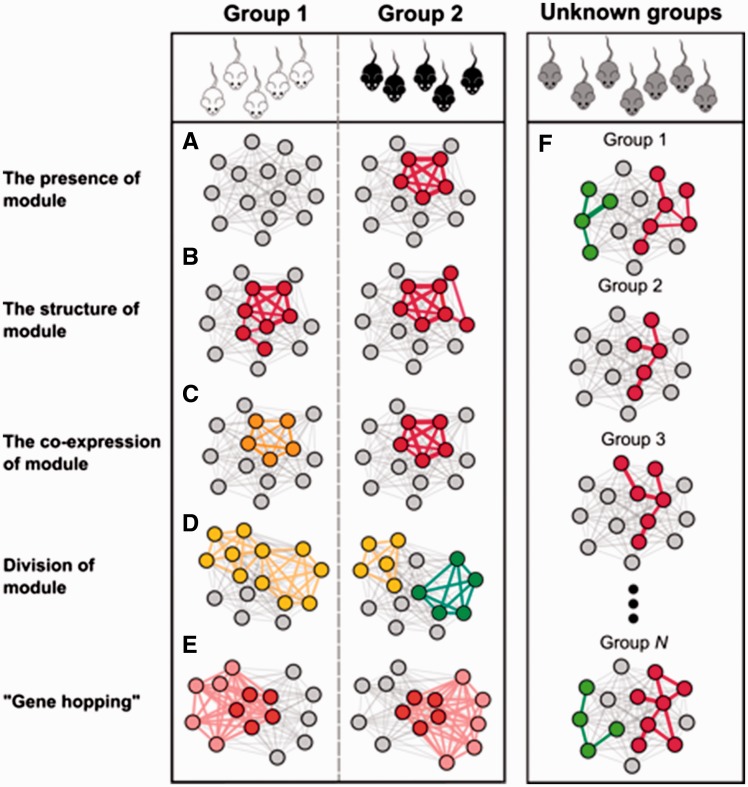
Changes in gene co-expression patterns that can occur between samples. Differential co-expression can occur as the presence of a module in only one of the sample groups (**A**), as differences in the structure of the module (**B**) or as differences in the correlation strength between members of the modules (**C**). Additionally, differential co-expression can be detected if one larger interconnected module splits into several smaller ones (**D**) or if a group of genes changes its correlation partners [‘gene hopping’ (**E**)]. If sample groups are not defined before the differential co-expression analysis, or are unknown, biclustering methods can identify modules unique to a subpopulation of samples by simultaneously classifying the samples into groups in which these modules exist (**F**).

WGCNA determines the activity and importance of each module in each subpopulation of samples (Figure 3A and 3C). For each module, an eigengene is calculated, which is the vector that best describes the expression behaviour (in a linear fashion) of all genes within this module in the samples included in the analysis. It then prioritizes which genes in these modules are likely to underlie the phenotype associated with the module by identifying either genes behaving similarly to the eigengene of the module or those genes that are intra-modular hub genes (these tend to coincide). By design, DICER is tailored to identify module pairs that correlate differently between sample groups, e.g. modules that form one large interconnected module in one group compared with several smaller modules in another (Figure 3D). DICER may be particularly useful for time series experiments in which co-expression changes are gradual, e.g. cell cycle series experiments, where modules are specific to a particular phase and co-expressed in transitions between phases. DiffCoEx focuses on modules that are differentially co-expressed with the same sets of genes. The most extreme case of this behaviour is sets of genes that ‘hop’ from one set of correlated genes to another in a coordinated manner (Figure 3E). In this case, DiffCoEx would cluster ‘hopping’ genes in a similar manner. DINGO is a more recent tool that works similarly to DiffCoEx by grouping genes based on how differently they behave in a particular subset of samples (representing e.g. a particular condition) from the baseline co-expression determined from all samples [102]. These are the most likely genes to explain different phenotypes that are associated with the two different networks. Each of the methods detects specific module changes by design, but they can also detect modular changes that they were not specifically designed for and may outperform other tools in the identification of these changes [130].

A number of studies have used differential co-expression network analyses to identify networks unique to specific tissues [11] or disease states [134]. The rapid increase in publicly available RNA-seq data and projects such as GTEx and ENCODE, which generate large-scale RNA-seq profiles, has enabled co-expression analysis within and across different tissues [11, 15]. The GTEx project collects and provides expression data from multiple human tissues for the study of gene expression, regulation and their relationship to genetic variation [135]. In a study comparing RNA-seq data from 35 tissues from the GTEx data set, a tissue hierarchy was constructed based on the average gene expression in each tissue. Related tissues, such as those from different brain regions, clustered together. This hierarchy was used to construct a single combined co-expression network derived from the tissue-specific co-expression networks—a meta-network. It was then shown that in tissue-specific networks, TFs with functions specific to that tissue tend to be highly expressed together with tissue-specific genes. These genes tend to form a stronger connection with each other than with other genes, but remain at the periphery of the network (thus having low centrality), while the tissue-specific TFs become more central to that module [11]. Thus, tissue-specific TFs could be uncovered by identifying modules with increased co-expression strength in tissue-specific networks (Figure 3A and 3C) and by pinpointing the central hubs of these modules. In contrast, genes that are not TFs but are tissue-specific should be detectable by identifying genes that are at the periphery in these modules (Figure 3B). Moreover, some TFs have different roles in different tissues. These TFs would be expected to be hub genes that are central to one module under one condition and central to another module in another condition.

Differentially connected genes are those with different co-expression partners between two sample groups. These genes appear to play a regulatory part in the difference in the phenotype observed between two groups (Figure 3D) [8–10]. For example, one study compared co-expression in mutant cattle with increased muscle growth with co-expression in non-mutants, using a method similar to DiffCoEx. By identifying the most differentially expressed genes and TFs showing the highest differential connection to these genes [10] (Figure 3D), the TF containing the causal mutation (myostatin) was identified. Interestingly, the *Mstn* gene, which encodes this TF, hardly changed in expression itself, providing an example of how differential co-expression analysis can uncover biologically important findings not revealed by differential expression analysis alone.

Not all methods construct a co-expression network to assess differential expression. GSNCA [103] can be used to identify differentially co-expressed gene sets, which have to be defined a priori, between two sample groups. In the first step this method determines weight vectors for each sample group, from a correlation network. These weight vectors represent the cross-correlation of each gene with all the other genes, effectively summarizing a correlation matrix into a single vector, describing a weight for each gene. These weights for the genes representing a certain gene set are then compared between two sample groups, to determine whether the gene set is differentially co-expressed.

#### Generalized Single Value Decomposition (GSVD)

Generalized Single Value Decomposition (GSVD) is a unique type of differential co-expression analysis that relies on spectral decomposition to identify modules of co-regulated genes. Unique to this approach is that it summarizes the expression of samples and all genes into a smaller number of variables, aiming to explain as much expression variation in as few variables as possible. Here we focus on the summary of gene expression into principal components or ‘genelets’, a term introduced in [104] that can be interpreted as an analogy to co-expressed modules, and which represent the partial expression of multiple genes. The relative significance of these genelets—describing the extent to which a signal from the genelet is present (that is, the extent to which the genelet is expressed) in a data set—can be compared between two data sets. If the significance is similar, the genelet represents a co-expression pattern shared between the two data sets, whereas differences in significance indicate that the co-expression pattern is unique to one of the data sets. Higher Order (HO)-GSVD was more recently developed and uses a similar approach for comparisons between more than two data matrices [105].

GSVD was first used in 2003 to analyse microarray expression data from human and budding yeast to identify common and unique pheromone and stress response patterns between these two species [104]. HO-GSVD recently proved effective at identifying pathways important for self-renewal of neural progenitors [136]. GSVD was shown to identify patterns unique to glioblastoma multiforme, a type of brain tumour, which was useful for prognostic purposes [137]. Similarly, genelets that are active in normal samples were identified [138]. These genelet signals were then removed from the total signal in cancer samples, revealing a cancer-specific signature [138]. Both of these studies demonstrated that signatures unique to the cancer had a strong signal for genes duplicated in the cancer [137, 138], as is common in cancers, suggesting that identified profiles reflect the oncogenic events in the genome.

It is not surprising that differential co-expression methods are growing in popularity as the cost of high-quality expression data decreases. While these methods have not yet been applied to RNA-seq data, recent findings from microarray studies make this an exciting prospect. However, because these methods are sensitive to outliers, they require high-quality data.

### Differential co-expression without prior grouping

An alternative method for detecting differentially expressed clusters between subpopulations of data is biclustering. If a data set contains several biologically distinct but unknown sample groups, biclustering can identify genes with a similar expression pattern in only a sub-set of the samples without the need for prior sample classification (Figure 3F). This is particularly useful when such information is not available, as can be the case for large-scale single-cell RNA-seq experiments like those using the Drop-seq system [139] or inDrop [140].

In a clinical study it is often possible to predefine groups of healthy and diseased samples. However, the same disease can manifest through different mechanisms. This is a scenario common in cancer, where different mutations can lead to different alterations in co-expression patterns but a similar phenotype [7]. Biclustering allows researchers to disentangle the mechanisms in the cases where predefining biologically relevant sample groups is difficult. For this purpose, biclustering is more effective than other co-expression analysis methods [7].

Cheng *et al.* were first to use biclustering in co-expression analysis [141], followed by the development and application of many more biclustering approaches (reviewed by Pontes et al. [106]). The choice of biclustering method depends on the number of samples and factors such as whether the samples are species- or tissue-specific and whether the included samples constitute disease phenotypes and/or different time points. Biclustering methods can be computationally challenging depending on the method used [106]. Methods should be selected carefully because different biclustering approaches can have varying results in the same data set [142].

Biclustering approaches were recently applied to RNA-seq-based expression data. Analysis of the expression data from several developmental stages of worm and fruit fly, by identifying biclusters containing similar orthologous gene sets unique to different developmental stages between the two species, led to the identification of genes with a similar, and thus conserved, function in development [132]. Biclustering has also been applied to single-cell RNA-seq data [5]. Because biclustering groups genes and samples simultaneously, it enabled the simultaneous identification of groups of cell types and corresponding gene modules to reveal 49 different cell types and their corresponding cell-type-specific gene modules, results that were later supported by experimental validation [5]. With the emergence of single-cell RNA-seq, biclustering methods may be able to identify cell-type-specific modules that are present in diseased but not in healthy cells.

Another biclustering method identified miRNAs deregulated in breast cancer through their presence in biclusters unique to cancer samples [7]. These miRNAs have been suggested as markers for diagnosis and treatment response [7]. Biclustering has also been used to identify tightly co-expressed sets of protein-coding genes unique to subpopulations of cancer patients, which could be used to understand patient prognosis and to further precision medicine approaches [18, 20]. In another cancer data set, a three-dimensional clustering method (triclustering) was used to identify genes co-expressed across subpopulations of samples and time points [6]. This method effectively identified several known breast cancer genes in a breast cancer cell line by identifying hub genes in triclusters differentially expressed between cancer samples at early and late time points using the eigengene changes between the samples of each tricluster [6].

### Comparison of differential co-expression analysis methods

While a comprehensive and unbiased comparison of methods used in differential co-expression analysis is desirable, the performance of the tools may be situation-dependent, varying between species, disease states and perhaps even data sets, thus making it difficult to identify the optimal method in each circumstance. An attempt was recently made to compare 10 differential co-expression algorithms, but concluded that it remains difficult to evaluate these owing to the lack of gold standard gene sets to validate the outcome of these methods [143]. Several of the tools described in this review have been compared in publications introducing a competing method. DICER has been argued to perform better than DiffCoEx and CoXpress [4] based on functional enrichment analysis of differentially expressed modules. HO-GSVD outperformed WGCNA and DiffCoEx based on its ability to detect clusters in simulated data [136]. Although biclustering is a powerful approach, it does not necessarily perform better than other network analysis methods such as WGCNA, as shown by a comparison using different tools on simulated data [144]. However, as discussed earlier, biclustering can be performed without the need for prior sample group classification.

Although many of the tools and methods described in this review were originally created for microarray data, they are also applicable to RNA-seq data. There are RNA-seq-specific differential co-expression analysis methods, harnessing the exon- or isoform-specific expression information or allele-specific expression effects, that have been reported to perform better than other tools where this information is not considered [52, 145]. However, it is unclear whether these differential co-expression methods also perform better if other methods are supplied with the same isoform-specific expression information, which could be determined before differential co-expression analysis. As a result, it remains difficult to assess whether these new tools perform better than already well-established tools such as WGCNA, which can also be used on isoform-specific expression data [50].

Because the tools described in this review create modules based on different criteria, it is also questionable whether the measures used in the comparisons represent desirable properties for all cases. For example, DiffCoEx groups genes based on their differential co-expression behaviour, whereas WGCNA identifies modules that are co-expressed in multiple samples and conditions. In a homogenous data set, DiffCoEx will likely detect fewer and smaller modules, indicating that there are not many differentially co-expressed genes. This will likely lead to lower enrichment scores when the performance of DiffCoEx is compared with WGCNA on such homogenous data, whereas it is merely an indication that not many co-expression partner changes occur in the data. By contrast, tools that focus on shared co-expression modules are likely to find strong correlation modules with high enrichment scores, which may not be relevant if the goal is to identify regulatory modules. WGCNA has been widely shown to perform well under many different circumstances and for different purposes [54]. However, it requires information on the sample conditions to assign modules to conditions. If this information is not available (as in large-scale single-cell RNA-seq experiments) or if researchers wish to identify subgroups within the sample groups, biclustering is a more suitable approach.

To get a systematic assessment of the performance of different tools and methods, projects such as DREAM4 and DREAM5 [146] have been invaluable. These challenged researchers to construct regulatory networks from simulated and *in vivo* benchmark data sets. As these challenges are predefined they allow researchers to test their methods/tools in an unbiased manner. However, these challenges were last posed in 2010 and many new methods and tools have been developed since.

## Integrated network analysis

Experimental validation often focuses on single genes. As these experiments are costly and time-consuming, high confidence predictions of causal genes are of great importance. An analysis based solely on co-expression does not (yet) provide this level of confidence. Therefore, incorporation of information from other types of data can help to prioritize genes that may underlie a phenotype. This can be achieved, for example, using information describing which genes are TFs, as is done for regulatory predictions by GENIE3 [113]. However, a focus on TFs is rarely sufficient, and integration of multiple data types is often required to increase the accuracy and usefulness of the resulting networks [13, 147].

### TF binding site analysis

Genome-wide transcription factor binding site (TFBS) analysis was introduced in the beginning of this millennium using chromatin immunoprecipitation followed by microarray analysis, also known as ChIP-chip [148], which was later replaced by the more accurate ChIP-seq [149]. These data were used to create a genome-wide integrated regulatory network from gene expression and TFBS data [150]. Combined analysis of ChIP-chip-based TFBSs and expression data initially showed that, in 58% of the cases, the TFs bound to the promoter region of the gene were indeed regulated by the corresponding TF [151]. A partial least squares approach (a well-known method for analysis of high-dimensional data with several continuous response variables) was later proposed to identify false positives and distinguish the activation and repression activities of TFs [152]. A more recent method harnesses the rapidly increasing availability of ChIP-seq data in combination with expression data to rank the genes bound by a TF, which can be used to prioritize the most likely TF targets [153]. Tools to conduct similar analyses, integrating expression and ChIP data, have also been published [154].

### Multilayer integrated networks

Independent from the approach used to identify them, network modules can be further investigated for shared eQTL gene targets, TF/miRNA targets or enriched binding motifs [15, 120]. Several computational methods and publicly available data sets are available for multi-omics data integration. For example, information about eQTLs can be acquired from recent large-scale blood-based *trans*-eQTL meta-analysis [155] or eQTL studies conducted in other tissue types [156]. Transcription factor binding sites (TFBSs) can be collected from databases such as JASPAR and DeepBind [157], which consist of TF binding motifs inferred from experimental data. Binding sites can be further prioritized by investigating tissue-specific ChIP-seq peaks from ENCODE [15]. Finally, miRNA–target interactions can be identified using several *in silico* target prediction tools [158, 159] or using manually curated databases of experimentally supported target interactions [160–162].

**Figure 4:**
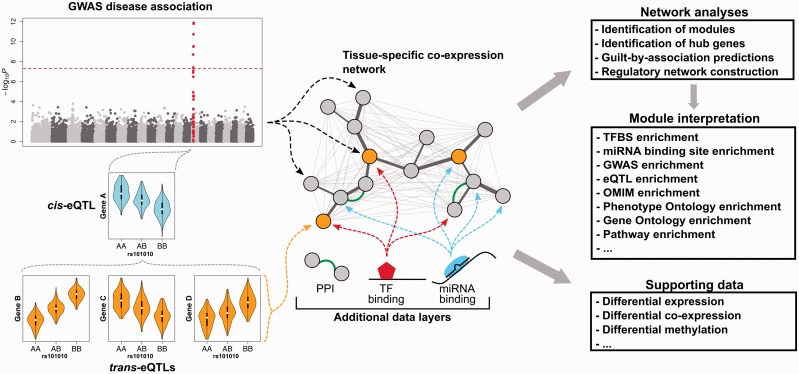
Strategies for integrating multi-omics data with co-expression analyses. Networks are more informative if they are constructed using expression data specific to the tissue of interest. Genomic variation can be mapped to a co-expression network either by linking suggestive GWAS hits to the genes in the network or by first identifying genetic variants with an effect on gene expression levels (*cis*- and *trans*-eQTLs) and then mapping those to the co-expression network. Additional data layers may include TFBSs (based on binding motifs or ChIP-seq/ChIP-chip experiments), miRNA target binding sites (based on *in silico* predictions or experimental techniques) and established protein–protein interactions. A co-expression network can be used to identify modules, hub genes and for predicting the function of unknown trait-associated genes. Identified modules can be analysed by enrichment analyses to identify overlaying features. Additionally, the research hypothesis can be supported by additional differential expression, co-expression and methylation analyses that can be performed if respective omics data are available for cases and controls for a corresponding trait. eQTL: expression quantitative trait loci; GWAS: genome-wide association study; OMIM: online Mendelian inheritance in man; miRNA: microRNA; PPI: protein–protein interaction; TF: transcription factor; TFBS: TF binding site.

Combining information from different layers of data may lead to new biologically interpretable associations in a number of ways. If intra-modular hub genes are TFs or targets of a TF, this TF is more likely to have a causal role in the phenotype under investigation [10]. If multiple Genome-Wide Association Study (GWAS) hits exist in the same module, their cumulative presence can significantly contribute to disease development [120, 163, 164]. Differential methylation states of genes within a co-expression module can elucidate methylation patterns underlying disease [165]. If multiple genes are regulated by the same genetic variant (under a *trans*-eQTL effect), it may be possible to identify the gene responsible for the alterations of the network by identifying the *cis*-eQTL gene driving the *trans*-eQTL effects (Figure 4). This is supported by the fact that genes under *trans*-regulation of disease-associated genomic variants are sometimes functionally connected with the processes or pathways associated with the corresponding disease. Good examples of this are IFN (interferon)-α and complement pathways in which several genes were under *trans*-regulation of a systemic lupus erythematosus-associated variant, possibly via *cis*-regulation of *IKZF1* [155]. The integration of regulatory genetic variant information into co-expression network analysis, with *cis*-eQTLs used as causal anchors, identified *TYROBP* as the most likely causal factor in late-onset Alzheimer disease patients, a finding supported by the observation that mutations in this gene are known to cause Nasu-Hakola disease [128]. Lastly, copy number variation can affect gene expression levels, and including such information may help identify and/or explain alterations in co-expression network structures present in diseases or traits [138].

Overall, integration of multiple data types increases the accuracy of the resulting predictions [13, 147]. For example, modules unique to different subtypes of cancer were identified by integrating tumour genome sequences with gene networks [166], and these modules may be useful for prognosis and identification of putative targets for personalized medicine-based treatments. A number of tools, described earlier in this review, can be used for differential co-expression analysis, but can also be applied to other data types. In the initial DINGO publication, the authors conducted a combined analysis on mRNA expression, DNA copy number variation and methylation data. By overlaying the differential networks of each data type and identifying edges present in all of them, a number of genes from the PI3K pathway were identified as important players in glioblastoma multiforme patients [102]. This pathway is an already-established therapeutic target, supporting the notion that this is an effective approach for identifying relevant targets for disease studies [167]. A recently published tool, CoRegNet, allows the integration of different types of data in a co-expression analysis by identifying co-operative regulators of genes from different data types [114]. Another established approach, cMonkey, achieves similar data integration by calculating the joint bicluster membership probability from different data types by identifying groups of genes that group together in multiple data types [115].

## Future prospects

In recent years, differential co-expression analyses have been increasingly used to analyse large data sets. This may be attributed to the decreased costs of large-scale gene expression profiling, in particular RNA-seq, to increased sample sizes, and to the greater availability of tissue-specific data from perturbation experiments, which are required for fruitful differential co-expression analyses [103, 168]. Likewise, biclustering algorithms have benefitted from larger sample sizes and higher data quality, as shown by the identification of co-expressed modules unique to cancer subtypes [18, 20]. The usefulness of biclustering on single-cell RNA-seq data has been demonstrated by the classification of different cell types and by the identification of clusters of genes uniquely co-expressed in specific cell types [5]. We expect these approaches to be more widely applied in the future, as they benefit from an increase in RNA-seq data quantity and quality, which will allow for more accurate identification of tissue-specific and cell-type-specific disease-related modules and regulators.

Large-scale single-cell sequencing technology is increasingly used and the first co-expression studies using such techniques have uncovered cell-type-specific co-expression modules that would have gone undetected in multi-cell-type co-expression analyses [5, 12]. Because the latter represent the aggregated signals of multiple cell types, they usually cannot detect alterations in cell subpopulations between different experimental groups. This is supported by the observation that the expression of cell cycle genes associated with ageing decreased in the analysis of non-cell-type-specific data [169]. However, data from single-cell experiments revealed that this observation was caused by a decreased proportion of the G1/S cells that highly express cell cycle genes rather than by altered expression across the whole cell population [170].

An additional prospect is the detection of mutations from RNA-seq data [171]. As mutations accumulate with age in different cells, these can be used to identify the origin of the cell. Mutation accumulation has been used to study cancer development and the origin of metastases [172]. In large-scale single-cell RNA-seq experiments, mutations could be used to separate cells based on their origin, or to group cells based on the mutations they harbour [173]. Cells harbouring the same mutations can be investigated for co-expression patterns, and modules unique to cells with a specific mutation may be detected. This may allow the direct linking of mutations to expression modules, with the limitation that only mutations in coding regions are detectable in RNA-seq data.

Although there are many exciting new possibilities with single-cell RNA-seq data, important challenges remain. Typically, a low number of reads per cell are sequenced and then the signal from multiple cells of the same type is aggregated to acquire a cell-type-specific gene-expression profile. It is hard to acquire sufficient data for rarer cell populations, such as stem cells, and this is currently limiting analyses on these cell types. Additionally, the low number of reads per cell leads to sparse expression matrixes to which normalization methods currently used in canonical RNA-seq analyses are not attuned. These normalization methods often also assume that the majority of genes do not change in expression between different samples, which is not necessarily the case in single-cell RNA-seq owing to variation in expression across different cells. This is further exacerbated by the difficulty in obtaining high-quality RNA from single cells. These and other issues are further discussed in [174].

In addition to the normalization issues that occur in single-cell RNA-seq, the optimal method for normalizing bulk RNA-seq data is also still not clear. The widely used Fragment/Reads Per Kilobase Million (FPKM) normalization has been debated [58] and although alternatives have and are being created, each method has its limitations. Additionally, from our experience, the use of different mapping tools can in some cases lead to different results. Although some comparisons between different tools and methods have been made [175], a large-scale comparison, using e.g. public data, would identify such cases and define best practices for pursuing each research question.

With the increased availability of different data types such as RNA-seq, genome sequences, ChIP-seq, methylome and proteome data, it will become possible to integrate these data sets to more accurately predict regulatory genes. Projects from large consortia like GTEx [156], the Epigenome Roadmap [176] and ENCODE [15] are already generating data from multiple-omics levels that facilitate these integrated analyses. To identify regulatory relationships, perturbation data are preferable, as canonical data cannot distinguish between true and false positives in regulatory relationships [129, 168]. Furthermore, regulatory relationships can be highly cell-type-, tissue- or developmental-stage-specific [129]. Only a handful of tools and methods are currently available to investigate multi-omics data, and the tools that exist mostly integrate only two layers of omics data [177]. Integrated network analyses come with additional mathematical challenges, and best practices are far from established. Further research on this topic is of great interest to the research community, as it will allow a better understanding of regulatory mechanisms that can explain co-expression patterns and disease mechanisms. A better understanding of these disease mechanisms and corresponding co-expression patterns will facilitate the identification of appropriate targets for intervention studies.

Key Points

- RNA-seq-based co-expression analysis can be used to assign putative functions to non-coding RNAs and to identify candidates for roles in disease.
- In co-expression networks, hub gene identification has a limited power for identifying targets for follow-up studies; yet, this can be enhanced by integrated network analyses, which may incorporate GWAS hits, eQTLs, TFBSs and other data layers.
- Differential co-expression analyses can reveal genes that have different co-expression partners between healthy and disease state and can help to uncover regulators underlying disease and other phenotypes.
- Methods such as biclustering and Generalised Single Value Decomposition (GSVD) allow the identification of signals/modules unique to specific cancer subtypes, which may serve a purpose in prognosis and for precision medicine.

## Notes

**Sipko van Dam** is a researcher at the Department of Genetics, UMC Groningen. He carried out his doctoral work at the University of Liverpool creating and analysing a co-expression network constructed from public RNA-seq data.

## Notes

**Urmo Võsa** is researcher in the Department of Genetics, UMC Groningen. His main interests lie in the genetics of gene expression and integration of data from different layers of genomic complexity to untangle the causes of complex diseases.

## Notes

**Adriaan van der Graaf** is a master’s student of Molecular Biology and Biotechnology at the Department of Genetics, UMC Groningen, focusing on novel statistical techniques in the analysis of expression data.

## Notes

**Lude Franke** is an associate professor at the Department of Genetics at the University Medical Centre Groningen. He is a statistical geneticist, working on analysing data on the genetics of complex and autoimmune diseases (e.g. celiac disease).

## Notes

**João Pedro de Magalhães** is a reader at the University of Liverpool where he leads the *Integrative Genomics of Ageing Group* (http://pcwww.liv.ac.uk/∼aging/). The group’s research integrates different strategies but its focal point is developing and applying experimental and computational methods that help bridge the gap between genotype and phenotype, and help decipher the human genome and how it regulates complex processes like ageing.

## Glossary

**Betweenness centrality**

This measure is used to describe the centrality (and therefore relevance) of a node in the network by counting the number of shortest paths between any other pair of nodes going through this node (Figure 2).

**Biclustering**

A clustering method that allows clustering based on two dimensions simultaneously, e.g. genes and different treatments.

**ChIP-chip**

This method identifies TFBSs by immunoprecipitation of the TF together with bound DNA fragments (chromatin immunoprecipitation—ChIP). A DNA microarray is subsequently used to identify the sequences where the corresponding TF is bound.

**ChIP-seq**

This method uses the same approach as ChIP-chip, but using RNA-seq rather than microarray to identify TFBSs.

**Clustering**

A statistical method of grouping variables. In the case of gene co-expression, genes that have similar expression patterns across multiple samples.

**Co-expression partner**

A gene whose expression shows a similar pattern across different samples to that of a gene of interest.

**Co-expression network**

A network that describes which genes have a tendency to show a coordinated expression pattern across a group of samples. In these networks, each node represents a gene and each edge represents the presence and/or the strength of the co-expression relationship.

**Eigengene**

A vector that best describes the expression changes of a module between different samples. This describes the partial expression of each gene (albeit to a different extent for each gene) in a linear fashion.

**Gene**

A DNA sequence that can be transcribed into a transcript. In the case of protein coding genes, this transcript can be translated into a protein. Proteins are the building blocks of our body. Non-coding genes are transcribed but do not encode proteins.

**Genelet**

Similar to the eigengene; a vector that represents the partial expression of multiple genes, but calculated using a different method. Unlike an eigengene, a genelet does not necessarily best explain the expression variability present in the data.

**Generalized single value decomposition**

A type of differential co-expression analysis that compares the strength of gene expression signatures existing in one group of samples with the strength for this signal in another group.

**Hierarchal clustering**

A clustering method that creates a hierarchical tree based on the distance between the nodes or genes in the network. Nodes that are close to each other in the network are part of the same branch. Clusters can be defined by cutting the branches at a certain height.

**Intra-modular hub**

A gene with high centrality within a module. These tend to have high biological relevance.

**Inter-modular hub**

A gene with high centrality in a network.

**k-means clustering**

A clustering method where a predefined number of clusters are constructed so that each gene is assigned to the cluster with the closest mean.

**Microarray**

A platform for quantifying gene expression that assays mRNA molecules based on their hybridization to probes present on an array, typically a glass slide.

**Module**

A group of co-expressed genes that form a sub-network in the larger network, usually defined by applying clustering algorithms on a co-expression network or directly on expression profiles.

**Mutual information**

The measure of dependence between two otherwise unrelated variables.

**Network robustness**

A measure of how resistant a network is to the removal of single nodes, assessed by the effect of removal on the connectivity of the network. The connectivity indicates how many nodes need to be removed to disconnect part of a network.

**Regulatory gene**

A gene that regulates the expression of other genes.

**Subpopulation-specific modules**

A co-expression module that only exists in a subset of the samples analysed.

**Transcript**

A single-stranded RNA molecule resulting from the transcription of a gene.

**Triclustering**

A clustering method that allows clustering in three dimensions simultaneously, e.g. genes, different treatments and time points.
